# Longitudinal Analysis of Substance Use Disorder Symptom Severity at Age 18 Years and Substance Use Disorder in Adulthood

**DOI:** 10.1001/jamanetworkopen.2022.5324

**Published:** 2022-04-01

**Authors:** Sean Esteban McCabe, John E. Schulenberg, Ty S. Schepis, Vita V. McCabe, Philip T. Veliz

**Affiliations:** 1 Center for the Study of Drugs, Alcohol, Smoking and Health, University of Michigan, Ann Arbor; 2 Institute for Social Research, University of Michigan, Ann Arbor; 3 Institute for Research on Women and Gender, University of Michigan, Ann Arbor; 4 Institute for Healthcare Policy and Innovation, University of Michigan, Ann Arbor; 5 Department of Psychology, University of Michigan, Ann Arbor; 6 Department of Psychology, Texas State University, San Marcos; 7 Department of Psychiatry, University of Michigan, Ann Arbor

## Abstract

**Importance:**

Although more than 1 in every 3 US individuals will develop a substance use disorder (SUD) in their lifetime, relatively little is known about the long-term sequelae of SUD symptoms from adolescence through adulthood.

**Objective:**

To evaluate the longitudinal associations between adolescents’ SUD symptom severity with later medical use of prescription drugs (ie, opioids, sedatives, and tranquilizers), prescription drug misuse (PDM), and SUD symptoms at ages 35 to 50 years.

**Design, Setting, and Participants:**

Eleven cohorts of US 12th grade students were followed longitudinally from age 18 years (1976-1986) to age 50 years (2008-2018) in the Monitoring the Future (MTF) study. Baseline surveys were self-administered in classrooms, and follow-ups were conducted by mail. Data were analyzed from June 2021 to February 2022.

**Exposure:**

Response to MTF study between 1976 and 2018.

**Main Outcomes and Measures:**

Sociodemographic variables were measured at baseline. All bivariate and multivariate analyses use attrition weights to adjust for attrition by age 50 years within the sample. SUD symptoms, prescription drug use, and PDM were measured at baseline and every follow-up.

**Results:**

The sample of 5317 individuals was 51.2% female (2685 participants; 95% CI, 49.6%-52.6%) and 77.9% White (4222 participants; 95% CI, 77.6%-79.1%). Participants were surveyed beginning at age 18 years and ending at age 50 years. The baseline response rate ranged from 77% to 84%, and the 32-year retention rate was 53%. Most adolescents with most severe SUD symptoms at age 18 years had 2 or more SUD symptoms in adulthood (316 participants [61.6%]; 95% CI, 55.7%-66.9%), and this association held for baseline alcohol, cannabis, and other drug use disorder symptoms. Adolescents with the highest SUD symptom severity at age 18 years had the highest adjusted odds of prescription drug use and PDM in adulthood (4-5 symptoms, adjusted odds ratio, 1.56; 95% CI, 1.06-2.32; ≥6 symptoms, adjusted odds ratio, 1.55; 95% CI, 1.11-2.16). The majority of adults using prescribed opioids, sedatives, or tranquilizers (568 participants [52.2%]; 95% CI, 48.4%-55.9%) in the past year had multiple SUD symptoms at age 18 years.

**Conclusions and Relevance:**

These findings suggest that most adolescents with severe SUD symptoms do not transition out of symptomatic substance use, and the long-term sequelae for adolescents with more severe SUD symptoms are more deleterious than those for adolescents with no or low severity. Prescribers should be aware that many adults prescribed opioids, sedatives, or tranquilizers had multiple SUD symptoms during adolescence and require careful assessment and monitoring.

## Key Points

**Question:**

What are the long-term sequelae of substance use disorder (SUD) symptoms from adolescence through adulthood?

**Findings:**

In this national multicohort study of 5317 individuals followed from ages 18 to 50 years, the majority of adolescents with the most severe SUD symptoms had 2 or more SUD symptoms in adulthood. Most adults using prescribed opioids, sedatives, or tranquilizers had multiple SUD symptoms during adolescence.

**Meaning:**

These findings suggest that most adolescents with severe SUD symptoms do not transition out of symptomatic substance use over a 32-year period, and prescribers must be aware that many adults prescribed controlled substances had SUD symptoms during adolescence and require careful assessment.

## Teaser

This cohort study evaluates the association between substance use disorder symptoms at adolescence and substance use disorder in adulthood using data from the Monitoring the Future study.

## Introduction

Drug overdose deaths are the leading cause of injury-related death in the US, accounting for more than 100 000 deaths in 2021.^1,2,3^ The increases in drug overdose deaths in recent years make it imperative to develop more effective efforts toward early identification of individuals who are at the greatest risk for developing substance-related consequences, such as overdose and substance use disorder (SUD). More than 1 in every 3 US individuals will develop an SUD in their lifetime, and the prevalence of SUD is highest during young adulthood.^4,5,6^

Early experiences from childhood and adolescence set the stage for adult functioning.^7,8,9^ Long-term trajectories of alcohol and cannabis use are relatively well documented from adolescence into young adulthood.^10,11,12,13,14,15^ In contrast, relatively little is known about the long-term sequelae of SUD symptoms from adolescence through adulthood. The associations between adolescents’ SUD symptom severity and later prescription drug use, prescription drug misuse (PDM), and SUD symptoms during adulthood deserve more attention.

The US Preventive Services Task Force recently concluded that “the current evidence is insufficient to assess the balance of benefits and harms of screening for unhealthy drug use in adolescents.”^16^ The current lack of information regarding the long-term sequelae of adolescent SUD symptoms represents a key knowledge gap with direct relevance for screening, diagnosis, prevention, and treatment efforts.^16^ The percentage of adolescents with more severe SUD symptoms who persist with SUD symptoms in adulthood remains unknown. The Monitoring the Future (MTF) study is a US national, longitudinal, multicohort study that assesses SUD symptoms over a 32-year period from ages 18 to 50 years. The primary objective of the present study was to examine the association between SUD symptoms during adolescence and the presence of later prescription drug use, PDM, and SUD symptoms in adulthood at ages 35, 40, 45, and 50 years.

## Methods

### Sample

This multicohort study used panel data from the MTF study.^17,18^ This study meets the Strengthening the Reporting of Observational Studies in Epidemiology (STROBE) reporting guideline. Because the present study used deidentified data, it was deemed exempt from review and the requirement for informed consent by the University of Michigan institutional review board.

MTF surveyed nationally representative samples of US high school seniors each year since 1975 using self-administered questionnaires. Parents received a waiver of informed consent, providing them a means to decline their child’s participation after receiving a complete description of the study. Approximately 2450 high school seniors (modal age 18 years) were randomly selected each year for biennial follow-ups and surveyed using mailed questionnaires through age 30 years. One random half of each cohort started biennial surveys at age 19 years, and the other random half started at age 20 years. From age 35 years to age 50 years, respondents were surveyed every 5 years (ie, ages 35, 40, 45, and 50 years).

Respondents were randomized to 1 of 6 survey forms at baseline (12th grade), and the baseline SUD symptom items were included on only 1 form. Thus, the analytical sample contained data from one-sixth of the 11 cohorts of high school seniors (1976-1986) who were surveyed at baseline. Follow-ups were conducted biennially from modal ages 19 or 20 years through 29 or 30 years and at modal ages 35, 40, 45, and 50 years, for a total of 10 follow-up surveys (data collection occurred from 1976 through 2018). The baseline response rates over the study period ranged from 77% to 84%, with most nonresponses due to absence (<1% refused to participate). The mean (SD) retention rate from first follow-up to age 50 follow-up (among those who could have made it to age 50) is 63.6% (10.9%). To help correct for potential attrition bias, and to be consistent with other MTF panel analyses,^13,19,20^ we incorporated attrition weights to account for respondent characteristics associated with nonresponse at follow-up. The MTF study design, protocol, and sampling methods are described in greater detail elsewhere.^17,18^

### Measures: Key Baseline Variables

SUD symptoms in adolescence (measured at baseline, age 18 years) were measured with several questions based on *Diagnostic and Statistical Manual of Mental Disorders* (*DSM*) criteria for alcohol use disorder (AUD), cannabis use disorder (CUD), and other drug use disorder (ODUD). Versions IV and 5 of the *DSM* were used over the course of the study. Fifteen items were used to assess whether respondents ever encountered problems as they relate to substance use, including failure to fulfill major role obligations, continued substance use despite persistent or recurrent interpersonal or social problems, and continued substance use when physically or psychologically hazardous. Each of the 15 items or symptoms were summed and recoded into 3 separated categorical variables (AUD, CUD, and ODUD) with the following categories: 0 symptoms, 1 symptom, 2 to 3 symptoms, 4 to 5 symptoms, and 6 or more symptoms. The SUD categorical variables were determined with the maximum number of symptoms for AUD, CUD, or ODUD symptoms. Reliability for these 15 items was considered strong according to Cronbach α scores (AUD, α = .813; CUD, α = .846; ODUD, α = .885) and resulted in estimates for categories closely resembling other national estimates.^4,5,6^

### Measures: Key Outcome Variables

Past-year medical prescription drug use was measured at ages 35, 40, 45, and 50 years with identical questions based on separate measures assessing past-12-month medical use of prescription opioids, sedatives, and tranquilizers. Respondents were asked on how many occasions (if any) they had taken a particular prescription medication class because a physician told them to take it. Respondents were provided a list of several generic and brand name examples for each of the prescription drug classes (eg, Vicodin, OxyContin, and codeine for prescription opioids; Ambien, Lunesta, and Sonata for prescription sedatives; and Librium, Valium, and Xanax for prescription tranquilizers). The response scales for each of the prescription drug use questions ranged from 1 for no occasions to 7 for 40 or more occasions. Each measure was recoded as a binary variable (no occasions vs any use). To assess any medical prescription drug use frequency (a combined measure of opioids, sedatives, and tranquilizers), respondents who indicated any prescription drug use were flagged as engaging in past-year medical prescription drug use; this was done separately at age 35, 40, 45, and 50 years.

Past-year PDM was measured at ages 35, 40, 45, and 50 years with identical questions based on separate measures assessing past-12-month misuse of prescription opioids, sedatives, and tranquilizers (ie, “…taken any…on your own—that is, without a doctor telling you to take them?”). As with medical prescription drug use, respondents were provided with generic and trade medication names, the response scale was identical, and each measure was recoded to be binary. To assess any PDM frequency (a combined measure of opioids, sedatives, and tranquilizers), respondents who indicated any type of PDM were flagged as engaging in past-year PDM; this was done separately at age 35, 40, 45, and 50 years.

SUD symptoms in adulthood (ages 35 to 50 years) were measured with similar items used at baseline concerning AUD, CUD, and ODUD over the past 5 years. The 15 items were consistent with SUD measurements in other large-scale surveys^21,22,23^ and reflect *DSM-IV* and *DSM-5* AUD, CUD, and ODUD symptoms.^13,20,24^ We followed recommended practice that each SUD was indicated by meeting 2 or more of the criteria,^25^ resulting in estimates closely resembling other national estimates.^4,5,6^

### Measures: Covariates

Sociodemographic variables and substance use behaviors at baseline (age 18 years) included: sex (men or women), race and ethnicity (Black, Hispanic, White, or other; other was defined as Asian, American Indian, those who selected multiple races or ethnicities, and those with missing racial information), geographical region (Northeast, Midwest, South, or West), urbanicity based on metropolitan statistical area (MSA; large MSA, 12-16 of the largest urban areas; other MSA, other cities or towns of at least 50 000 inhabitants; or non-MSA, fewer than 50 000 inhabitants), parental education (neither parent graduated from a 4-year college or at least 1 parent graduated from a 4-year college), average grade in high school (C+ or lower or B− or higher), cohort year, past-month cigarette use, past 2-week binge drinking, and past-month marijuana use. Race and ethnicity options were defined by the MTF study team. Race and ethnicity were assessed in this study because of differences across behavioral and social outcomes. Completion of a 4-year college degree or higher by age 50 was also included in the fully adjusted models.

### Statistical Analysis

Descriptive statistics and unadjusted odds ratios were generated to examine bivariate associations between number of SUD symptoms at age 18 years and (1) the past-5-year prevalence of 2 or more SUD symptoms at ages 35 to 50 years, and (2) the prevalence of past-year PDM at ages 35 to 50 years. Second, logistic regression models were fitted using the generalized estimating equations method^26,27^ with an exchangeable correlation structure to assess the association between the number of SUD symptoms at age 18 years and the past 5-year prevalence of SUD symptoms or past-year PDM during this 15-year period (ages 35-50 years) in middle adulthood when accounting for the key covariates. All bivariate and multivariate analyses use attrition weights to adjust for attrition by age 50 years within the sample. Significance tests were set at the .05 alpha level (two-tailed). Statistical analyses were completed using Stata statistical software version 17.0 (StataCorp). Data were analyzed from June 2021 to February 2022.

## Results

At baseline, 5317 12th graders were in the longitudinal sample. Among the respondents, 51.2% (2685 participants; 95% CI, 49.6%-52.6%) were female, and 77.9% (4222 participants; 95% CI, 77.6%-79.1%) were White. Approximately 1 in 5 respondents (20.1%; 95% CI, 18.9%-21.3%) indicated 2 to 3 SUD symptoms, 12.1% (697 participants; 95% CI, 11.1%-13.0%) reported 4 to 5 SUD symptoms, and 11.5% (753 participants; 95% CI, 10.6%-12.4%) reported 6 or more SUD symptoms at age 18. Table 1 shows the descriptive statistics associated with the sociodemographic characteristics of the analytic sample.

**Table 1.** Sample Characteristics and Substance Use Disorder Symptom Severity at Age 18 Years

Sample characteristics at age 18 y	Participants, No. (%) [95% CI] (N = 5317)^a^
Baseline variables (12th grade)	
Sex^b^	
Male	2630 (48.8) [47.3-50.3]
Female	2685 (51.2) [49.6-52.6]
Race and ethnicity^c^^,^^d^	
Black	566 (12.3) [11.3-13.2]
Hispanic	197 (3.8) [3.2-4.3]
White	4222 (77.9) [77.6-79.1]
Other^e^	332 (6.1) [5.4-6.8]
Grade point average^f^	
B– or higher	3692 (72.6) [71.2-73.9]
C+ or lower	1534 (27.4) [26.0-28.7]
Parental education level^g^	
Less than college degree	3359 (66.1) [64.6-67.5]
College degree or higher	1742 (33.9) [32.4-35.3]
Urbanicity or MSA^d^^,^^h^	
Large MSA	1412 (25.4) [24.1-26.7]
Other MSA	2227 (42.1) [40.6-43.4]
Non-MSA	1678 (32.5) [31.1-33.9]
US region^d^	
Northeast	1285 (23.5) [22.2-24.7]
Midwest	1581 (29.3) [28.0-30.6]
South	1598 (31.4) [30.1-32.8]
West	853 (15.8) [14.7-16.8]
Cohort year^d^	
1976-1978	1410 (26.3) [25.0-27.5]
1979-1981	1458 (26.6) [25.3-27.8]
1982-1984	1477 (27.7) [26.4-29.0]
1985-1986	972 (19.4) [18.2-20.5]
AUD symptoms at age 18 y^i^	
No symptoms	1866 (46.5) [46.5-12.4]
1	593 (12.4) [11.4-13-4]
2-3	1070 (21.6) [20.3-22.9]
4-5	576 (11.5) [10.5-12.4]
≥6	458 (8.0) [7.2-8.8]
CUD symptoms at age 18 y^j^	
No symptoms	2735 (69.6) [68.2-71.0]
1	432 (8.1) [7.3-8.9]
2-3	625 (10.9) [9.9-11.8]
4-5	345 (5.3) [4.6-5.9]
≥6	380 (6.1) [5.4-6.8]
ODUD symptoms at age 18 y^k^	
No symptoms	3633 (90.3) [89.4-91.1]
1	236 (3.7) [3.2-4.3]
2-3	214 (3.0) [2.5-3.4]
4-5	97 (1.2) [1.0-1.4]
≥6	142 (1.9) [1.5-2.2]
SUD symptoms at age 18 y^l^	
No symptoms	1981 (46.3) [44.8-47.8]
1	498 (10.0) [9.1-10.9]
2-3	1090 (20.1) [18.9-21.3]
4-5	697 (12.1) [11.1-13.0]
≥6	753 (11.5) [10.6-12.4]

Abbreviations: AUD, alcohol use disorder; CUD, cannabis use disorder; MSA, metropolitan statistical area; ODUD, other drug use disorder; SUD, substance use disorder.

^a^ Unweighted sample sizes are provided. Percentages are weighted to adjust for the oversampling of drug users into the panel sample. At baseline, 5317 twelfth graders were selected into the longitudinal sample between 1976 and 1986 who completed Form 3 of the Monitoring the Future (MTF) study. It should be noted that 1907 respondents did not complete any follow-up between ages 35 and 50 years.

^b^ The missing data percentage for the demographic characteristic sex was 0.4%.

^c^ Race and ethnicity options were defined by the MTF study team and included: Hispanic, non-Hispanic Black, non-Hispanic White, and other.

^d^ Other was defined as Asian, American Indian, those who selected multiple races or ethnicities, and those with missing racial information.

^e^ Missing data percentages for race and ethnicity, urbanicity or MSA, region, and cohort year were 0.0%.

^f^ The missing data percentage for grade point average was 3.0%.

^g^ The missing data percentage for parent education level was 4.4%.

^h^ Metropolitan statistical areas (MSA) were defined as: Large MSA (12–16 of the largest urban areas), Other MSA (other cities/towns of at least 50 000 inhabitants), and Non-MSA (fewer than 50 000 inhabitants).

^i^ The missing data percentage for AUD was 16.1%.

^j^ The missing data percentage for CUD was 16.3%.

^k^ The missing data percentage for ODUD was 19.3%.

^l^ The missing data percentage for composite SUD was 6.5%.

As shown in Table 2, the bivariate associations among alcohol, cannabis, and other drug use disorder symptom severity at age 18 years and subsequent prescription drug use, PDM, and SUD symptoms in adulthood (ages 35-50 years) were positive across the baseline symptom severity subgroups (ie, no symptoms, 1 symptom, 2-3 symptoms, 4-5 symptoms, and ≥6 symptoms). Those having 2 or more SUD (composite) symptoms at baseline had higher odds (compared with those with no symptoms) of past-year medical prescription drug use and of PDM in adulthood, whereas those having 1 or more SUD (composite) symptoms at baseline had higher odds of having 2 or more SUD (composite) symptoms in adulthood. The majority of adolescents with most severe SUD symptoms (6 or more [composite score]) at age 18 (316 participants [61.6%]; 95% CI, 55.7%-66.9%) had at least 2 SUD (composite) symptoms in adulthood, and this association held for baseline AUD symptoms (198 participants [63.9%]; 95% CI, 56.5%-70.5%), CUD symptoms (168 participants [64.2%]; 95% CI, 55.8%-71.7%), and ODUD symptoms (64 participants [64.1%]; 95% CI, 49.0%-77.3%).

**Table 2.** Prevalence of Past-Year Prescription Drug Use, Misuse and Substance Use Disorder Symptoms in Adulthood by Substance Use Severity at Age 18

Symptoms	Percentage (95% CI])^a^	OR (95% CI)^b^		Percentage (95% CI])^a^	OR (95% CI)^b^		Percentage (95% CI])^a^	OR (95% CI)^b^	Percentage (95% CI])^a^		OR (95% CI)^b^
AUD symptoms at age 18	Medical prescription drug use			Prescription drug misuse			≥2 SUD symptoms		≥2 AUD symptoms		
No. of participants	2903		NA	2902		NA	2904	NA	2896	NA	
No symptoms	15.7 (13.5-18.1)	1 [Reference]		11.1 (9.2-13.3)	1 [Reference]		(29.3 (26.3-32.3)	1 [Reference]	27.5 (24.6-30.4)		1 [Reference]
1	19.1 (15.1-23.6)	1.25 (0.91-1.74)		15.8 (12.3-19.9)	1.49 (1.05-2.12)		46.1 (40.4-51.7)	2.06 (1.57-2.70)	44.2 (38.7-49.9)		2.09 (1.59-2.75)
2-3	22.7 (19.5-26.1)	1.57 (1.21-2.02)		17.4 (14.6-20.6)	1.69 (1.26-2.26)		54.2 (49.9-58.4)	2.85 (2.28-3.57)	50.8 (46.5-55.1)		2.72 (2.18-3.41)
4-5	26.9 (21.6-32.7)	1.97 (1.41-2.74)		23.8 (18.7-29.8)	2.50 (1.73-3.62)		61.2 (55.1-66.8)	3.80 (2.85-5.06)	57.8 (51.6-63.6)		3.61 (2.71-4.81)
≥6	23.2 (18.3-28.9)	1.62 (1.14-2.28)		21.9 (17.0-27.5)	2.24 (1.54-3.24)		63.9 (56.5-70.5)	4.26 (3.04-5.96)	61.9 (54.6-68.6)		4.28 (3.07-5.97)
Linear trend (AUD 0-15)	NA	1.07 (1.03-1.11)		NA	1.10 (1.06-1.15)		NA	1.23 (1.18-1.29)^c^	NA		1.22 (1.17-1.28)
CUD symptoms at age 18	Medical prescription drug use			Prescription drug misuse			≥2 SUD symptoms		≥2 CUD Symptoms		
No. of participants	2858		NA	2857		NA	2859	NA	2802	NA	
No symptoms	17.2 (15.3-19.1)	1 [Reference]		12.6 (10.9-14.3)	1 [Reference]		32.8 (30.4-35.3)	1 [Reference]	5.2 (4.1-6.5)		1 [Reference]
1	27.3 (20.6-34.9)	1.80 (1.22-2.65)		22.1 (16.0-29.7)	1.97 (1.28-3.03)		54.1 (46.3-61.5)	2.40 (1.73-3.33)	16.2 (11.1-22.9)		3.54 (2.14-5.84)
2-3	24.2 (19.8-29.0)	1.53 (1.15-2.04)		22.6 (18.3-27.5)	2.03 (1.50-2.76)		66.8 (61.2-71.9)	4.12 (3.15-5.38)	19.5 (15.4-24.2)		4.43 (3.04-6.46)
4-5	21.7 (16.1-28.3)	1.33 (0.91-1.95)		21.2 (15.7-27.7)	1.86 (1.26-2.75)		68.2 (59.4-75.9)	4.39 (2.94-6.57)	21.8 (15.4-30.1)		5.15 (3.12-8.47)
≥6	24.5 (18.8-31.0)	1.55 (1.09-2.22)		22.3 (16.7-29.2)	2.01 (1.35-2.97)		64.2 (55.8-71.7)	3.66 (2.54-5.28)	23.1 (17.4-30.1)		5.53 (3.57-8.56)
Linear trend (CUD 0-15)	NA	1.05 (1.01-1.09)		NA	1.11 (1.06-1.15)		NA	1.26 (1.20-1.33)^c^	NA		1.23 (1.18-1.29)
ODUD symptoms at age 18 y	Medical prescription drug use			Prescription drug misuse			≥2 SUD symptoms		≥2 ODUD symptoms		
No. of Participants	2700		NA	2699		NA	2701	NA	2688	NA	
No symptoms	17.8 (16.1-19.5)	1 [Reference]		13.8 (12.2-15.4)	1 [Reference]		39.5 (37.2-41.8)	1 [Reference]	6.2 (5.1-7.3)		1 [Reference]
1	24.2 (17.3-32.3)	1.46 (0.95-2.24)		21.7 (15.3-29.9)	1.74 (1.10-2.73)		60.0 (49.6-69.3)	2.28 (1.49-3.49)	18.0 (11.5-26.9)		3.34 (1.93-5.80)
2-3	30.8 (22.6-40.3)	2.05 (1.33-3.17)		28.5 (20.1-37.9)	2.45 (1.54-3.91)		68.5 (58.6-77.0)	3.34 (2.14-5.20)	18.5 (12.4-26.2)		3.44 (2.09-5.64)
4-5 symptoms	44.4 (30.7-58.0)	3.62 (2.02-6.47)		46.7 (33.0-60.5)	5.46 (3.05-9.79)		73.3 (59.3-84.7)	4.36 (2.21-8.57)	28.9 (17.8-42.9)		6.16 (3.22-11.7)
≥6	35.2 (24.5-47.6)	2.51 (1.48-4.26)		34.1 (22.9-47.6)	3.26 (1.84-5.78)		64.1 (49.0-77.3)	2.77 (1.46-5.27)	23.1 (14.9-34.6)		4.65 (2.59-8.34)
Linear trend (ODUD 0-15)	NA	1.13 (1.07-1.20)		NA	1.18 (1.11-1.26)		NA	1.20 (1.09-1.32)^c^	NA		1.21 (1.14- 1.29)
SUD symptoms at age 18 y	Medical prescription drug use			Prescription drug misuse			≥2 SUD symptoms		NA		NA
No. of participants	3192		NA	3191		NA	3193	NA	NA		NA
No symptoms	16.1 (14.0-18.5)	1 [Reference]		11.2 (9.3-13.2)	1 [Reference]		27.9 (25.1-30.8)	1 [Reference]	NA		NA
1	19.3 (14.9-24.5)	1.23 (0.87-1.75)		13.5 (9.9-18.1)	1.24 (0.83-1.86)		38.8 (32.8-45.0)	1.63 (1.22-2.19)	NA		NA
2-3	21.9 (18.8-25.2)	1.45 (1.13-1.86		16.1 (13.4-19.1)	1.52 (1.14-2.03)		54.0 (49.8-58.1)	3.03 (2.43-3.77)	NA		NA
4-5	24.4 (19.8-29.6)	1.67 (1.22-2.29)		23.0 (18.4-28.1)	2.36 (1.68-3.32)		60.0 (54.3-65.4)	3.88 (2.95-5.10)	NA		NA
≥6	23.8 (19.7-28.3)	1.62 (1.21-2.15)		23.4 (19.2-28.2)	2.43 (1.77-3.35)		61.6 (55.7-66.9)	4.13 (3.13-5.45)	NA		NA
Linear trend (SUD 0-15)	NA	1.06 (1.03-1.09)		NA	1.11 (1.07-1.14)		NA	1.21 (1.17-1.26)^c^	NA		NA

Abbreviations: AUD, alcohol use disorder; CUD, cannabis use disorder; NA, not applicable; ODUD, other drug use disorder; OR, odds ratio; SUD, substance use disorder.

^a^ Percentages and 95% CIs are based on aggregated prevalence rates between ages 35 and 50 years.

^b^ Unadjusted ORs (no covariates) were estimated using generalized estimating equation binary logistic regression models with an exchangeable correlation structure.

^c^ Linear trend for each type of SUD was estimated separately with respect to the categorical analog and was treated as a continuous variable that ranged from 0 to 15.

Table 3 provides results of the multivariable controlled analyses. As shown, adolescents with the highest SUD symptom severity (composite measure) at age 18 years had roughly one and a half times higher odds of past-year prescription drug use in adulthood compared with those without SUD symptoms at age 18 years (4-5 symptoms, adjusted odds ratio, 1.56; 95% CI, 1.06-2.32; ≥6 symptoms, adjusted odds ratio, 1.55; 95% CI, 1.11-2.16). Similarly, the adjusted odds of past-year PDM and 2 or more SUD symptoms during adulthood were approximately 2 times greater among adolescents with 4 to 5 SUD and 6 or more SUD symptoms (composite score) at age 18 years when compared with those without SUD symptoms at age 18 years. Additionally, Table 3 also presents estimates (based on age of follow-ups) and shows that prescription drug use and PDM increased between age 35 years to age 50 years, whereas SUD symptoms decreased between ages 35 and 50 years. As shown in the Figure, the majority of adults prescribed opioids (275 participants [50.0%]; 95% CI, 44.7%-55.2%), sedatives (256 participants [53.9%]; 95% CI, 48.2%-59.6%), tranquilizers (340 participants [55.1%]; 95% CI, 50.0%-59.9%), or at least 1 of these classes (568 participants [52.2%]; 95% CI, 48.4%-55.9%) in the past year had reported 2 or more SUD symptoms at age 18 years.

**Table 3.** Multivariate Logistic Regression Results: Adult (Ages 35-50 Years) Prescription Drug Use, Misuse, and Substance Use Disorder Symptoms Adjusting for Covariates^a^

Variable	AOR (95% CI)		
	Prescription drug use	Past-year PDM	≥2 AUD symptoms
AUD			
Participants, No.	2628	2627	2624
AUD at age 18 y, symptoms			
No symptoms	1 [Reference]	1 [Reference]	1 [Reference]
1	1.12 (0.76-1.66)	1.16 (0.82-1.65)	1.50 (1.15-1.97)
2-3	1.33 (1.00-1.77)	1.39 (1.02-1.87)	1.69 (1.35-2.12)
4-5	1.72 (1.17-2.53)	1.98 (1.34-2.91)	2.14 (1.61-2.85)
≥6	1.53 (1.05-2.23)	1.91 (1.33-2.76)	2.68 (2.00-3.60)
Age, y			
35	1 [Reference]	1 [Reference]	1 [Reference]
40	1.26 (1.03-1.55)	1.25 (0.99-1.57)	0.74 (0.65-0.83)
45	1.64 (1.34-2.02)	1.59 (1.28-1.98)	0.72 (0.63-0.82)
50	1.89 (1.53-2.34)	1.52 (1.18-1.96)	0.59 (0.51-0.69)
AUD at age 18 y^b^			
Linear trend (AUD)	1.06 (1.02-1.10)	1.08 (1.04-1.12)	1.13 (1.09-1.16)
Linear trend (age)	1.24 (1.16-1.32)	1.16 (1.07-1.25)	0.85 (0.81-0.89)
ODUD	Prescription drug use	Past-year PDM	≥2 ODUD symptoms
Participants, No.	2420	2419	2408
ODUD at age 18 y, symptoms			
No symptoms	1 [Reference]	1 [Reference]	1 [Reference]
1	1.15 (0.74-1.79)	1.79 (1.12-2.87)	2.41 (1.37-4.25)
2-3	1.58 (1.03-2.42)	1.83 (1.16-2.87)	2.18 (1.32-3.60)
4-5	2.96 (1.73-5.06)	4.31 (2.44-7.60)	4.37 (2.16-8.81)
≥6	2.79 (1.59-4.90)	3.30 (1.90-5.73)	3.62 (2.13-6.15)
Age, y			
35	1 [Reference]	1 [Reference]	1 [Reference]
40	1.19 (0.96-1.49)	1.13 (.889-1.45)	0.64 (0.49-0.84)
45	1.52 (1.22-1.88)	1.52 (1.20-1.91)	0.67 (0.49-0.89)
50	1.76 (1.41-2.19)	1.54 (1.18-2.01)	0.54 (0.38-0.75)
ODUD at age 18 y^a^			
Linear trend (ODUD)	1.14 (1.08-1.21)	1.17 (1.11-1.23)	1.18 (1.12-1.24)
Linear trend (age)	1.21 (1.13-1.30)	1.17 (1.08-1.27)	0.82 (0.73-0.92)
CUD	Prescription drug use	Past-year PDM	≥2 CUD symptoms
Participants, No.	2587	2586	2542
CUD at age 18 y, symptoms			
No symptoms	1 [Reference]	1 [Reference]	1 [Reference]
1	1.53 (0.94-2.51)	1.74 (1.11-2.72)	3.02 (1.63-5.53)
2-3	1.33 (0.97-1.80)	1.77 (1.26-2.49)	2.85 (1.74-4.64)
4-5	1.31 (0.86-2.03)	1.78 (1.14-2.77)	3.44 (1.75-6.64)
≥6	1.51 (1.02-2.25)	1.68 (1.10-2.56)	3.41 (1.91-6.06)
Age, y			
35	1 [Reference]	1 [Reference]	1 [Reference]
40	1.25 (1.01-1.54)	1.26 (0.99-1.61)	0.46 (0.36-0.59)
45	1.54 (1.25-1.91)	1.56 (1.23-1.98)	0.33 (0.25-0.44)
50	1.75 (1.42-2.16)	1.62 (1.24-2.12)	0.28 (0.20-0.39)
CUD at age 18^a^			
Linear trend (CUD)	1.05 (1.00-1.10)	1.07 (1.03-1.12)	1.15 (1.09-1.22)
Linear trend (age)	1.20 (1.13-1.28)	1.17 (1.09-1.27)	0.62 (0.56-0.69)
SUD	Prescription drug use	Past-year PDM	≥2 SUD symptoms
Participants, No.	2867	2866	2867
SUD at age 18 y, symptoms			
No symptoms	1 [Reference]	1 [Reference]	1 [Reference]
1	1.10 (0.71-1.70)	1.01 (.671-1.52)	1.24 (0.93-1.67)
2-3	1.24 (0.94-1.65)	1.31 (.959-1.80)	1.89 (1.51-2.38)
4-5	1.56 (1.06-2.32)	2.08 (1.41-3.06)	2.16 (1.63-2.87)
≥6	1.55 (1.11-2.16)	1.97 (1.39-2.80)	2.62 (2.00-3.43)
Age, y			
35	1 [Reference]	1 [Reference]	1 [Reference]
40	1.20 (0.98-1.47)	1.18 (.945-1.48)	0.73 (0.66-0.81)
45	1.52 (1.24-1.85)	1.53 (1.24-1.90)	0.71 (0.63-0.79)
50	1.77 (1.45-2.17)	1.57 (1.23-2.01)	0.59 (0.51-0.67)
SUD at age 18^a^			
Linear trend (SUD)	1.06 (1.02-1.10)	1.09 (1.05-1.13)	1.12 (1.09-1.16)
Linear trend (age)	1.21 (1.14-1.29)	1.17 (1.09-1.26)	0.85 (0.81-0.89)

Abbreviations: AOR, adjusted odds ratio; AUD, alcohol use disorder; CUD, cannabis use disorder; ODUD, other drug use disorder; PDM, prescription drug misuse; SUD, substance use disorder.

^a^ All models were adjusted for covariates including baseline substance use (past 2-week binge drinking and past 30-day cigarette and marijuana use), US region, urbanicity, cohort year, race and ethnicity, sex, highest level of parental education, grade point average in high school, and whether the respondent ever completed college.

^b^ Linear trend for AUD, CUD, ODUD, SUD, and age were estimated separately with respect to the categorical analog: SUD was treated as a continuous variable that ranged from 0 to 15; age was treated as a continuous variable that ranged from 0 to 3.

**Figure.**
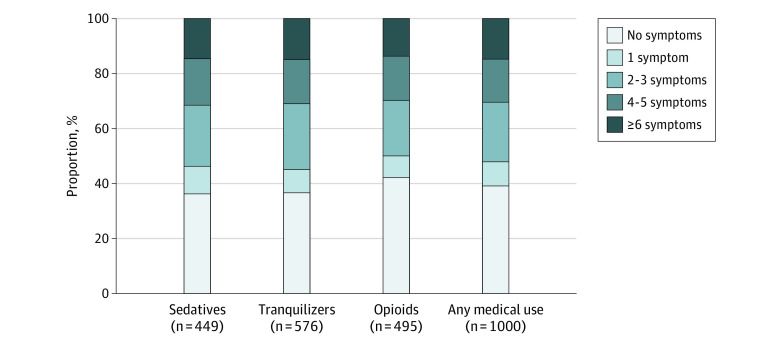
Past-Year Medical Use of Prescription Sedatives, Tranquilizers, and Opioids in Adulthood (Ages 35-50 Years) by Substance Use Disorder Symptoms at Age 18 Years

## Discussion

To our knowledge, this cohort study was the first national investigation to examine the long-term association of SUD symptoms in adolescence with prescription drug use, PDM, and SUD symptoms in adulthood. We found clear evidence for long-term associations of moderate to severe levels of SUD symptoms in adolescence with prescription drug use, PDM, and SUD symptoms in adulthood. The findings suggest that screening that accounts for SUD symptom severity can enhance identification of individuals at the greatest risk for later PDM and SUD.

The majority of US adolescents with the most severe levels of SUD symptoms reported having at least 2 SUD symptoms in adulthood, which differed considerably from those with 1 or fewer SUD symptoms. This finding reinforces that the long-term sequelae are more deleterious for those with more severe SUD symptoms during adolescence. Notably, most adolescents with severe SUD symptoms do not mature out of symptomatic substance use during the transition from adolescence to adulthood. Continued education of adolescents and young adults can promote healthy relationships with prescription drugs and other substances to prevent substance use during adolescence in an attempt to mitigate SUD through adulthood.

US adolescents with SUD symptoms were more likely to report prescription drug use and PDM in adulthood. The most severe SUD symptoms persist in adulthood; approximately one-fourth of these adults report prescription drug use involving prescription benzodiazepines, opioids, and sedatives, which has important clinical implications since being prescribed these controlled substances with such an SUD history is associated with an increased risk of PDM and SUD.^28,29^ Indeed, the majority of adults who reported past-year prescription drug use involving prescription benzodiazepines, opioids, and sedatives had symptomatic substance use during adolescence, raising questions about the safety of prescribing controlled substances to these individuals. Given that prescription drug use and PDM increased from ages 35 to 50 years for the overall sample, our findings regarding the long-term associations of adolescent SUD symptoms reinforces the importance of careful screening and medication monitoring when prescribing controlled substances. Importantly, the cohorts of adolescents who were high school seniors between 1976 to 1986 and were followed over the 32-year study time frame (ending 2018) runs parallel to the emergence and steep increase in the opioid crisis, largely associated with the widespread overprescribing of opioids.^30,31^ These prescribing practices may be associated with the high rates of prescription drug use and PDM as these cohorts aged into middle adulthood. Although the association between adolescent SUD symptom severity and adult prescription drug use and PDM would still stand, it is important to acknowledge that the opioid crisis has led to changes in prescribing practices over the past several years that warrant more research that reexamines these associations over time.

The long-term sequelae of adolescents with higher SUD symptom severity compared with those with lower SUD symptom severity may be indicative of distinct causal mechanisms contributing to individuals who are more predisposed to greater SUD symptoms. A number of reviews have asserted that severe SUD symptoms during adolescence may produce robust, long-term changes in neurobiological pathways and circuits that lead to persistent SUD symptoms and other disorders that lead to the higher prevalence of prescription medication use.^32,33,34,35^ Severe SUD symptoms may be initiated by or exaggerated by comorbid psychiatric disorders as well as family history of SUD.^36,37,38,39^ The contributing mechanisms are likely not mutually exclusive and together may amplify disease status. Because of the higher rates of psychiatric comorbidity among those with multiple *DSM-5* SUDs and the more persistent course of multiple SUDs, a greater emphasis toward identifying SUD severity and comorbid psychiatric disorders is warranted. The distinct characteristics and causal mechanisms of more severe SUD symptoms should be further investigated to (1) improve understanding of vulnerability to chronic and persistent SUD, (2) enhance screening and education, (3) identify potential points of early intervention, and (4) improve affordable and effective treatment.

The age-adjusted drug overdose death rate in the US has more than tripled over the past 2 decades, leading to more than 100 000 drug overdose deaths in the past year, which was the largest number of drug overdoses for a 12-month period ever recorded.^1,2,3^ There has also been a substantial shift nationally in the profile of individuals entering US substance abuse treatment facilities.^40,41,42^ Moreover, previous evidence from national surveys suggests that the prevalence of multiple SUDs among US adults with prescription drug use disorders increased considerably between 1991 to 1992 to 2001 to 2002.^43^ Taken together, these findings stress the importance of screening and accounting for polysubstance use as well as SUD severity during adolescence when identifying individuals who are at the greatest risk for SUD.

### Strengths and Limitations

The present study had several strengths and limitations that should be taken into account while considering implications of these findings. The MTF represents the first multicohort national panel study to assess SUD symptoms over a 32-year period from adolescence to age 50 years with consistent procedures and measures over time. The limitations included the exclusion of some subpopulations with higher rates of severe SUD symptoms, including school dropouts and institutionalized populations such as inmates currently in jails and prisons, which may have led to underestimation.^18,44^ It is worth highlighting that the estimated prevalence of adolescents in the present study who had AUD symptoms was comparable to the estimated prevalence in other national estimates.^5^ Although more research is needed to determine the characteristics of nonrespondents in national longitudinal substance use studies, several studies have found that attrition was higher among individuals with heavier substance use and more severe SUDs.^45,46,47^ Moreover, individuals with persistent and severe AUDs are more likely to die or be institutionalized than those who remit, resulting in selective survival.^45^ Additional analyses confirmed that adolescents with 6 or more symptoms at age 18 years had the lowest retention at age 50 years and were less prevalent than other national studies.^5^ The present study likely underestimates individuals with the most severe SUDs and inflates estimates of individuals with less severe SUD symptoms in the later intervals. The measures of SUD symptoms in adolescence and adulthood cannot establish formal *DSM*-based diagnoses given the study methods.

## Conclusions

The current study suggests that the presence of severe SUD symptoms during adolescence does not represent a temporary phase that most people age out of as they transition into adulthood. Importantly, the majority of adolescents with severe SUD symptoms persisted with 2 or more SUD symptoms and were more likely to report prescription drug use and misuse in adulthood. Most adults prescribed controlled substances had multiple SUD symptoms during adolescence. The present findings suggest that clinical assessment should screen for SUD symptom severity during adolescence and that continued education is warranted.
